# Molecular diversity, population structure, and linkage disequilibrium in a worldwide collection of tobacco (*Nicotiana tabacum *L.) germplasm

**DOI:** 10.1186/1471-2156-13-18

**Published:** 2012-03-21

**Authors:** Agostino Fricano, Nicolas Bakaher, Marcello Del Corvo, Pietro Piffanelli, Paolo Donini, Alessandra Stella, Nikolai V Ivanov, Carlo Pozzi

**Affiliations:** 1 Parco Tecnologico Padano, via Einstein, Loc. C.na Codazza, 26900 Lodi, Italy; 2 Philip Morris International R&D, Philip Morris Products SA, Quai Jeanrenaud 5, 2000 Neuchâtel, Switzerland; 3 Bayer CropScience, Technologiepark 38, 9052 Zwijnaarde, Belgium; 4 Fondazione Edmund Mach, 38010 San Michele all'Adige, TN, Italy

## Abstract

**Background:**

The goals of our study were to assess the phylogeny and the population structure of tobacco accessions representing a wide range of genetic diversity; identify a subset of accessions as a core collection capturing most of the existing genetic diversity; and estimate, in the tobacco core collection, the extent of linkage disequilibrium (LD) in seven genomic regions using simple sequence repeat (SSR) markers. To this end, a collection of accessions were genotyped with SSR markers. Molecular diversity was evaluated and LD was analyzed across seven regions of the genome.

**Results:**

A genotyping database for 312 tobacco accessions was profiled with 49 SSR markers. Principal Coordinate Analysis (PCoA) and Bayesian cluster analysis revealed structuring of the tobacco population with regard to commercial classes and six main clades were identified, which correspond to "Oriental", Flue-Cured", "Burley", "Dark", "Primitive", and "Other" classes. Pairwise kinship was calculated between accessions, and an overall low level of co-ancestry was observed. A set of 89 genotypes was identified that captured the whole genetic diversity detected at the 49 loci. LD was evaluated on these genotypes, using 422 SSR markers mapping on seven linkage groups. LD was estimated as squared correlation of allele frequencies (*r^2^*). The pattern of intrachromosomal LD revealed that in tobacco LD extended up to distances as great as 75 cM with *r^2 ^*> 0.05 or up to 1 cM with *r^2 ^*> 0.2. The pattern of LD was clearly dependent on the population structure.

**Conclusions:**

A global population of tobacco is highly structured. Clustering highlights the accessions with the same market class. LD in tobacco extends up to 75 cM and is strongly dependent on the population structure.

## Background

*Nicotiana tabacum *is a non-obligatory, selfing amphidiploid species derived from a hybridization event between *Nicotiana sylvestris *and *Nicotiana tomentosiformis *(summarized in [1]). As with other crops, breeding history and yield management have contributed to its genetic erosion [2].

Most of the existing variability is maintained at the *ex-situ *U.S. Nicotiana Germplasm Collection [3], which contains around 1,900 accessions of *N. tabacum*, including 656 cultivars and 1,244 tobacco introductions (TIs). The TIs probably capture most of the genetic variability that existed before modern agricultural intensification [2]. Before modern breeding [4], main tobacco classes were distinguished, based on method of curing and biochemical characteristics, into Flue-cured, Burley, Oriental, Cigar, Dark (air/fire cured), and Primitive. Burley tobaccos are believed to derive from a mutation identified in 1864 in a strain of Maryland tobacco, and Flue-cured are closely related to Dark fire-cured tobaccos [2].

To capture most of the genetic diversity with the least number of genotypes, subgroups out of larger populations of unrelated lines (core collections) are conveniently set up. Core collections have been assembled based on several algorithms [5-8] in several crops, including durum and bread wheats [5,6], barley [7], potato [8], maize [9], peanut [10], and rice [11]. The usage of molecular markers as descriptors of population structure provides the most reliable criteria when assembling core collections [12].

Linkage disequilibrium (LD) is defined as the non-random association of alleles at two or more loci. In cultivated plants, its extent is influenced by mating system, mutation rate, genetic drift, selection, recombination rate, gene conversion, and population size and structure [13]. Recently, LD has been used in association mapping [14] and to locate quantitative trait loci (QTLs) or major genes, based on the co-segregation of specific marker alleles and traits [15]. LD mapping has the potential to outperform traditional mapping because in a random-mating population over several generations, only close linkage between markers and traits remains, thus facilitating fine mapping. High-density genome fingerprinting could unveil long- and short-range LD. In the first case, in species with large genomes, a lower number of molecular markers can be tested [16], although this will result in a lower mapping resolution. Conversely, short-range LD enables the fine mapping of causal polymorphisms, if large panels of markers are available [17].

Data on the existence and extension of LD in different plant species are not exhaustive and point to a diversified picture, with decays of 1-2 kb in maize, up to 50 cM in Arabidopsis, and more than 50 cM in barley cultivars [18] although lower extent of LD have been reported in this species in collections of wild barley [19]. Most of the studies have been carried out in Arabidopsis and maize (summarized in [20] and [21], respectively), but data are available also for rice [18], aspen [22], loblolly pine [23], barley [24], wheat [25], grape [26], sugar beet [27], and soybean [28]. For the *Solanaceae *family, studies have been conducted in tomato [29] and potato [30].

The development of simple sequence repeat (SSR) markers has improved the characterization and use of genetic variation in *N. tabacum *[31]. SSRs have been adopted to evaluate genetic diversity in a tobacco collection by Moon et al. [3] and to study, in a collection of "Flue-cured" tobaccos, the changes in genetic diversity occurring over the last 70 years [32]. These studies prove the feasibility of using molecular markers to reconstruct the population structure in tobacco and represent the conceptual starting point for our study.

The aims of this study were to:

1) Assess the phylogeny and the population structure of 312 tobacco accessions representing a wide range of genetic diversity

2) Identify a subset of accessions as a core collection capturing most of the existing genetic diversity

3) Estimate, in the tobacco core collection, the extent of LD in seven genomic regions using SSR markers.

## Results and discussion

### Clustering of tobacco accessions based on SSR markers largely reflects their typological classification

A set of 312 pure lines derived from worldwide accessions of *N. tabacum *(Table 1) was investigated to detect the allelic variants at 49 SSR loci (Table 2). This panel of SSRs was selected based on technical reliability, uniqueness, and even distribution in the tobacco genome, as described in two papers by Bindler et al. [31,33], and was used to infer phylogeny and genetic diversity in the set of accessions, eventually leading to the assembly of a core collection.

**Table 1:** Geographical origin of the accessions considered

	Origin	Country	Number of accessions
Africa		Unspecified	1

		Ethiopia	1

		Malawi	4

		South Africa	2

		Zimbabwe	4

America	South America	Unspecified	5

		Argentina	6

		Brazil	6

		Peru	1

	Central America & Caribbean	Colombia	8

		Costa Rica	7

		Cuba	4

		Dom. Rep.	2

		Ecuador	2

		El Salvador	2

		Honduras	2

		Mexico	14

		Venezuela	10

	North America	Canada	1

		U.S.A.	69

Eurasia	Europe		

		Bulgary	3

		England	1

		France	1

		Germany	8

		Greece	9

		Holland	1

		Hungary	1

		Poland	3

		Spain	2

		Switzerland	2

		Ex-Yugoslavia	5

	India		2

	Russia		1

	Middle East	Iran	2

		Syria	1

		Turkey	15

	Far East	China	1

		Japan	3

		Taiwan	1

	Southeast Asia	Ceylon	1

		Indonesia	1

		Korea (Peninsula)	1

		New Guinea	1

		Philippines	2

Unknown			93

Total			312

**Table 2:** Genetic diversity in 312 tobacco accessions analyzed at 49 SSR loci distributed on seven linkage groups

SSR name	Na^a^	Nr^b^	PIC value^c^	*H_e_^d^*
PT50069	6		0.341493	0.362

PT60824	10	2	0.563837	0.629

PT61056	11		0.69481	0.729

PT54015	4	1	0.349554	0.425

PT51151	5		0.487583	0.570

PT61373	6		0.580958	0.623

PT52002	3		0.529461	0.602

PT50529	5	2	0.34733	0.398

PT50539	11	1	0.795385	0.819

PT51123	10	5	0.617252	0.660

PT54346	3		0.38183	0.500

PT51148	6	1	0.456057	0.504

PT52718	7	1	0.719666	0.760

PT51491	7		0.524293	0.577

PT53444	8	2	0.384821	0.408

PT54231	5	1	0.49717	0.535

PT61434	5		0.35199	0.374

PT50943	7	1	0.653921	0.692

PT51191	4		0.657827	0.710

PT51199	4		0.605402	0.671

PT53802	5		0.466876	0.561

PT55402	5	1	0.562008	0.634

PT1118n	8	3	0.607261	0.668

PT20275	9	1	0.567486	0.627

PT61336	13		0.588434	0.628

PT20388n	5		0.597306	0.664

PT1245	8	1	0.738271	0.774

PT51063	4		0.389199	0.435

PT51644	5	2	0.219817	0.236

PT52780	10	2	0.735723	0.761

PT53216	11		0.848799	0.863

PT55333	8	2	0.740353	0.776

PT61044	9		0.822021	0.841

PT53269	4		0.661365	0.712

PT53801	5		0.431335	0.533

PT54203	3	2	0.013311	0.013

PT606483	5		0.458545	0.552

PT50936	6		0.673446	0.724

PT51214	5	2	0.419422	0.517

PT51878	8	3	0.679454	0.725

PT50182	9	1	0.734941	0.769

PT51050	7	3	0.407602	0.516

PT54871	8	2	0.57878	0.606

PT52378	5		0.400721	0.466

PT52641	4		0.554108	0.621

PT1069n	11	2	0.709769	0.732

PT20224n	10	4	0.55947	0.628

PT50647	8		0.341428	0.356

PT53303	10		0.75943	0.788

Total	335 6.8 (avg.)			0.59 (avg.)

^a^Number of alleles

^b^Number of rare alleles (<5%)

^c^Polymorphic Index Content

^d^Gene diversity at each locus

The total number of alleles amplified at the 49 SSR loci was 335, with an average call rate of 99%. The high level of polymorphism revealed for the 49 SSR supported their usefulness for applications in diversity analysis.

The mean number of alleles detected for each locus was 6.84 (s.d. = 2.57), ranging from 13 alleles for marker PT61336 to three alleles for PT54203 and PT52002 (Table 2). This value is about half that recorded in previous studies [3,32]. The difference may be due to the choice of the marker loci as well as to the set of accessions analyzed. All tobacco accessions were genotyped as homozygous at the 49 SSR loci (*H_o _*= 0 at all loci, Table 2), with a gene diversity (*H_e_*) per locus spanning from 0.013 to 0.841 (average 0.59), a value lower than those reported in similar investigations carried out on TI accessions of tobacco [3]. The relatively low levels of *Hd *revealed by molecular markers in tobacco [34] can be due to relatively recent evolutionary and breeding bottlenecks, through which only a small proportion of the variability of the gene pools of the progenitor species was funneled through [2]. The polymorphic index contents (PIC) was > 0.4 at almost all loci, with PT53216 and PT54203 having the highest and lowest values, respectively (Table 2).

### Tobacco structured populations

**Figure 1:**
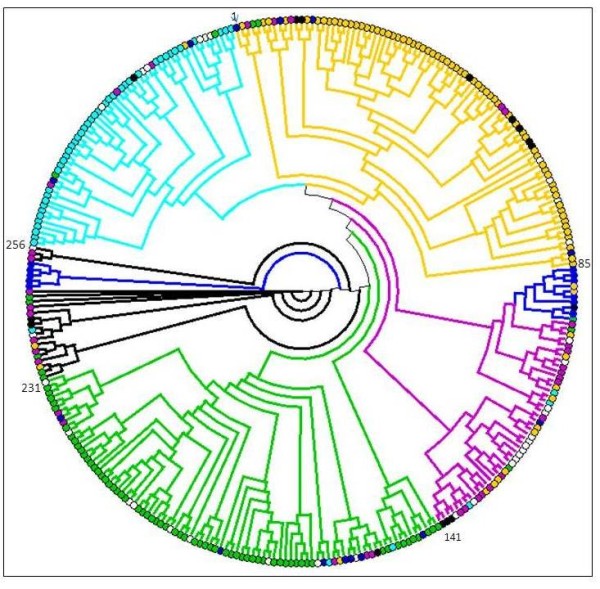
**Unrooted phylogenetic tree of 312 tobacco accessions constructed on the basis of 49 microsatellites loci using Cavalli-Sforza's genetic distance and the neighbor-joining method**. Clades represent accessions defined as "Burley" (light blue), "Primitive" (blue), "Dark" (black), "Cigar" (violet), "Oriental" (green) and "Flue-Cured" (yellow). Accession of "Other" and unknown types are shown as white circles. Clades are color coded according to the predominant tobacco type included, and when this was not possible, they are indicated with black lines. Numbers refers to the order in the list of the 312 accessions, provided as online Additional file 1.

Clustering of the 312 genotypes (Figure 1) revealed the relationships among accessions were distributed over six main clades. Accessions of "Oriental" clustered mainly in two different clades encompassing 88 accessions (green clades in Figure 1). Only 14 of the accessions were members of the heterogeneous group of tobacco accessions defined as "Other", whereas another 10 were classified as different tobacco types. "Flue-Cured" lines clustered mainly in one clade (yellow clade in Figure 1), although this also contained two, seven, four, and eight genotypes classified as "Oriental", "Dark", "Primitive", and "Other", respectively. Excluding six genotypes assigned to different clades, "Burley" accessions clustered in one clade (light-blue clade in Figure 1), which also contained nine members of the "Other" tobacco type and six lines classified as "Primitive", "Dark", "Oriental", and "Flue-cured". Non-group associated genotypes ("Other" in Table 1) clustered in two different clades (violet clades in Figure 1), one of which also included lines containing a large sub-set of the "Primitive" accessions (blue clade in Figure 1).

**Figure 2:**
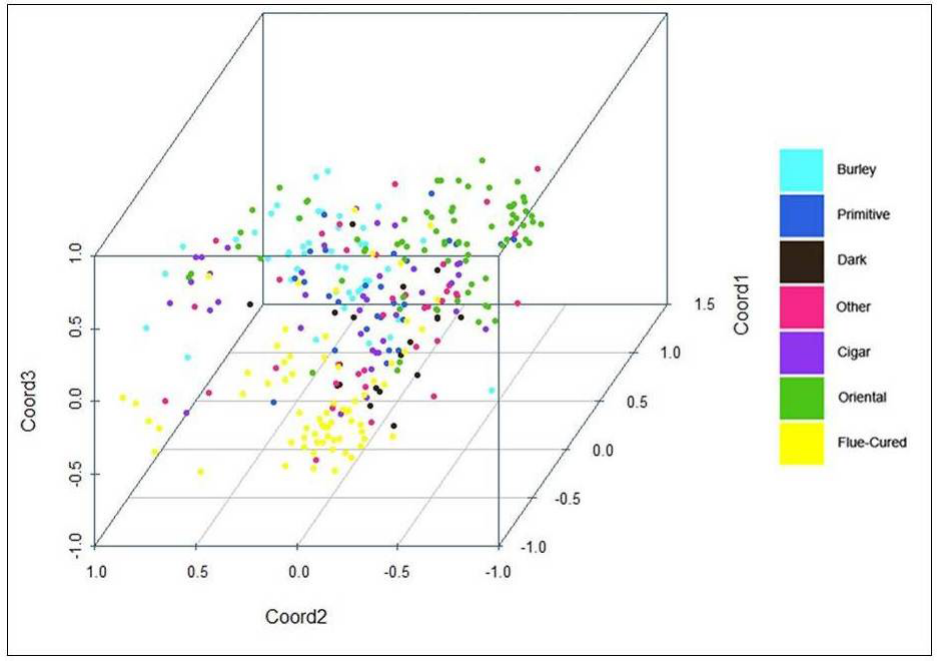
**Scatter-plot of the first three principal coordinates of PCoA considering data obtained from 49 SSRs**. Different colors indicate different tobacco types. Tobacco accessions of unknown type are not plotted.

Different tobacco types originated as the early growers saved seeds for subsequent planting, before the initiation of science-based breeding [3]. Tobacco growers selected plants for cultivation in different environments, for their agronomic performance using different agronomic practices, for the smoking characteristics of the leaf, and for adapting the leaf type to different leaf curing methods (i.e., the way the leaf is dried in a controlled way). The tobacco accessions we investigated clustered, based on molecular markers, according to their type, thus supporting the effectiveness of the breeding programs which have restricted the original breeding pool when selecting specifically for each market destination. The results are in agreement with previous data [31] supporting the correlation between type classifications and genetic distances [35]. The accessions that were found "contaminating" the homogeneity of groups (for example, "Cigar" varieties interspersed among "Oriental" varieties, in Figure 2), may be the result of misclassifications, as reported for the TI accessions [36]. In addition, inaccurate sampling procedures carried out during tobacco cultivation, or errors during varietal reproduction and conservation, can be the origin of the observed heterogeneity of major phylogenetic clades.

The distinct and homogeneous clustering of "Oriental" and "Flue-cured" tobaccos, the most outstanding tobacco types, is most likely due to the ~400 years of divergent selection in Europe/Middle East for the "Oriental" types [37], and to the adoption of a stringent conservative breeding strategy for "Flue-cured" tobaccos [38]. In "Flue-cured" tobaccos, genetic variability decreased significantly with the adoption of an "advanced cycle pedigree breeding", i.e., the exclusive usage of elite materials to produce breeding crosses [32]. Also "Burley" genotypes clustered together, although less homogenously than the previous two groups, as described also in [39], possibly because their selection has been traditionally performed on a wider geographic scale. Two phylogenetic clades were heterogeneous, containing most of the "Primitive" accessions, and the majority of "Cigar", "South American", and "Indonesian" tobaccos. This may represent the most ancient gene pool, making it particularly interesting for future breeding and mapping programs. According to the phylogeny, the "Primitive" genotypes should be genetically strongly related to "Cigar" tobaccos.

**Figure 3:**
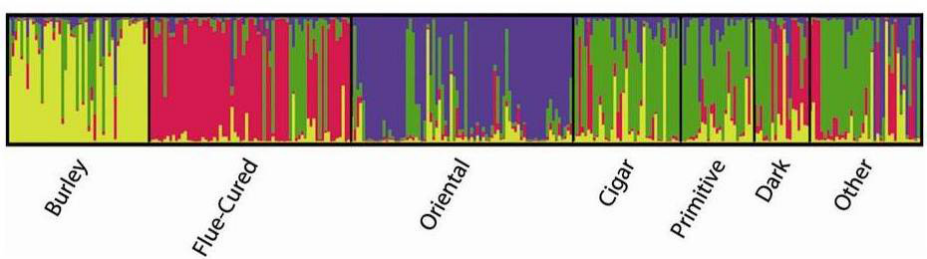
**Clustering of the 312 tobacco accessions according to a model-based Bayesian algorithm implemented in the program STRUCTURE**. Population memberships (expressed as%) for each accession are shown as estimates based on hypothetical subpopulations (see Methods). Each bar in the graph represents a single accession and its inferred proportion of admixture. The colors represent four different clusters corresponding to inferred unstructured subpopulations. The "Other" group includes genotypes of unknown type.

Principal coordinate analysis (PCoA) was carried out on the same SSR data set (Figure 2). The first principal component explained 40% of the genetic variance, and 71% was explained by the first three principal components, indicating that despite the high number of alleles detected at some SSR loci (Table 2), the collection was characterized by a narrow genetic basis. PCoA clustering indicated that molecular associations mainly reflected the physio-morphological characteristics associated with the tobacco types and their agronomic and commercial uses (Figure 2). A further Bayesian cluster analysis [40] identified the most probable number of *K *subpopulations present in the whole panel. The analysis of posterior probabilities supported the conclusion that four subpopulations had the highest likelihood (Figure 3). In the collection, a small number of genotypes were molecularly not aligned with their assignment to a tobacco type. Namely, while most of the "Burley", "Oriental", "Flue-cured", and "Cigar" genotypes clustered molecularly in four distinct subpopulations (yellow, violet, red, and green bars, respectively in Figure 3), "Primitive" and "Dark" genotypes were characterized by a more heterogeneous genome constitution. The close link between "Dark" and "Flue-cured" [2] was evidenced by the number of common alleles (red bars, Figure 3).

The levels of admixture (i.e., interbreeding between individuals of previously isolated populations) estimated by STRUCTURE appeared low in all lines considered, supporting the role of the conservative breeding to which the species was subjected.

Our PCoA results support the conclusion that the main tobacco types can be discriminated by molecular fingerprinting. In this sense, genetic distance and model-based analyses provide for the first time strong evidence of population substructure in tobacco.

### Kinship analysis reveals co-ancestry among burley tobaccos

To test the hypothesis of co-ancestry between tobacco accessions belonging to the same tobacco type, the pairwise kinship coefficients between accessions, as well as the population mean kinship (*MK) *among groups of tobacco accessions (Table 3), were calculated. The kinship coefficient is the ratio of the probability that, at a given locus, alleles of *i *and *j *individuals are identical by descent vs. the same probability of two random individuals. In this work alleles at one SSR locus were defined identical by descent if identical by state in the capillary electrophoresis analyses. Kinship coefficients are expressed relative to the average of the population and thus can assume negative values. The pairwise computations were used to calculate the *MK *coefficients in accessions of the same tobacco type, and in all possible pairwise combinations of the seven tobacco types (Table 3). The *MK *coefficient of the whole collection was -0.004326 revealing a generally low level of co-ancestry. When *MK *coefficient calculation was restricted to accessions of the same tobacco type, higher values of *MK *were obtained (Table 3). The highest value was obtained for the "Oriental" subset, while the lowest was obtained for the "Other" subset (both in bold in Table 3). The *MK *values calculated within types were positive, suggesting that a certain level of co-ancestry linked the accessions included in each tobacco type (Table 3).

**Table 3:** Population mean kinship coefficients (*MK*) calculated within and between groups of accessions from different tobacco types (in bold, the highest and lowest values; see text)

	BURLEY	CIGAR	DARK	FLUE CURED	ORIENTAL	PRIMITIVE	OTHER
**BURLEY**	0.05901750	-0.00520813	-0.00153207	-0.00805086	-0.03299411	-0.00642158	-0.01104828

**CIGAR**		0.01353207	-0.00020439	-0.01653192	-0.01012077	0.00785769	0.00308158

**DARK**			0.04352909	0.01887781	-0.03837019	-0.00021201	-0.01353367

**FLUE CURED**				0.05168934	-0.05120176	-0.02006903	-0.01492823

**ORIENTAL**					**0.07539302**	-0.01244403	0.00278055

**PRIMITIVE**						0.07323105	0.03543639

**OTHER**							**0.01063263**

### A set of 89 out of 312 genotypes captures the whole genetic diversity detected at 49 SSR loci

The first core collection of tobacco was created that identified the minimum set of accessions capturing most of the genetic diversity at the microsatellite loci tested on the full set of accessions. Five different lists of accessions selected using different rationales were created. The first list identified the minimum set of accessions capturing all 335 alleles identified in the whole panel of tobacco, and allowed us to isolate 60 genotypes. The other lists (60 genotypes each) were manually created based on Bayesian clustering, PCoA scatter-plot, co-ancestry analysis, and phylogeny. The five sets of accessions were then merged and a core collection was produced (Table 4) composed of 12 "Burley" (including 1 "Maryland"), 20 "Flue-cured", 20 "Oriental", 14 "Cigar", 10 "Primitive", 8 "Dark", and 5 "Other". Twenty-one of the accessions included in the core collection corresponded, according to Moon et al. [3], to samples collected before 1938. They still represent the best available sampling of the genetic diversity existing before modern breeding. Some of the genotypes (<5%) were selected because of their potential for tobacco breeding and not because they were identified following the protocols described.

**Table 4:** Tobacco accessions selected for the core collection

Name	Type	Origin
American Burley 1	BURLEY	US

Adiyaman	ORIENTAL	Turkey

Ambalema	CIGAR	Venezuela

Aparecido	PRIMITIVE	Venezuela

Bafra	ORIENTAL	Turkey

Banket A1	BURLEY	Zimbabwe

Barbasco	CIGAR	Ecuador

Basma Xanthi BX2A	ORIENTAL	Unknown origin

BB16A	BURLEY	France

BHAVYA	FLUE CURED	India

Big Cuba(i)n	CIGAR	Cuba

Basma 1	ORIENTAL	Unknown origin

Basma 2	ORIENTAL	Greece ?

Bonanza	PRIMITIVE	Mexico

BS 92	BURLEY	China

BU 21	BURLEY	USA

Cash	CIGAR	Mexico

Harmanlisjska Basma	ORIENTAL	Bulgaria

Chiricano	OTHER	Costa Rica

Chocoa	CIGAR	Colombia

COKER 347	FLUE CURED	USA

COKER 371 Gold	FLUE CURED	USA

Coltabaco 1A	DARK	Colombia

Cordoba	FLUE CURED	Mexico

Criollo	DARK	Costa Rica

Criollo Colorado	DARK	Argentina

Criollo especial	DARK	Cuba

Delcrest	FLUE CURED	Unknown origin

Deli (Sumatra)	PRIMITIVE	Honduras

BLACK MAMMOTH	DARK	Unknown origin

Dixie Bright 27	FLUE CURED	USA

Djebel 81 C	ORIENTAL	Unknown origin

Dreta	BURLEY	Germany

Dubek Nr 7	ORIENTAL	Poland

Dungowan	ORIENTAL	Unknown origin

Dynes	FLUE CURED	Australia

Florida 301	CIGAR	USA

Florida Sumatra	CIGAR	USA

South American Dark 1	CIGAR	South America

Gober Peloes	CIGAR	Brazil

Havana 322	CIGAR	USA

Hevesi 17	FLUE CURED	Hungary

Hicks Broadleaf	FLUE CURED	USA

Ilopango	PRIMITIVE	El Salvador

Itztepeque	PRIMITIVE	Costa Rica

K326	FLUE CURED	USA

K399	FLUE CURED	USA

Kabakulak Zagliveri	ORIENTAL	Unknown origin

Karabaglar	ORIENTAL	Turkey

Indonesian 1	OTHER	Indonesia

Indonesian 2	OTHER	Indonesia

Indonesian 3	OTHER	Indonesia

KDH-960 (TC 466)	DARK	USA

Komotini	ORIENTAL	Greece?

LITTLE CRITTENDEN	DARK	USA

LN German	CIGAR	Germany

Lonibow	FLUE CURED	Taiwan/Canada

MARYLAND 402	BURLEY	USA

McNair 135	FLUE CURED	USA

Mpeskq	ORIENTAL	Ex-Yugoslavia

NC 2326 (TC 365)	FLUE CURED	USA

Nevrokop 261	ORIENTAL	Mexico

Okinawa	PRIMITIVE	Japan

Orinoco	PRIMITIVE	Mexico ?

Oxford 207	FLUE CURED	USA

Payta	BURLEY	Unknown origin

PERIQUE (TC 556)	OTHER	Costa Rica?

TI 675	PRIMITIVE	Honduras ?

TI 1031	CIGAR	Venezuela?

Piyanguy Minas	FLUE CURED	Brazil

Saade 6	ORIENTAL	Unknown origin

Samsum Maden	ORIENTAL	Turkey

Saturn 280	BURLEY	Unknown origin

Sevilla 6	BURLEY	Spain

SIMMABA	DARK	Philippines

Speight 168	FLUE CURED	USA

TI 102 (Tobaco Negro)	CIGAR	Venezuela

TI 1271	ORIENTAL	Ethiopia

TI 1406	BURLEY	Germany

TI 592	PRIMITIVE	Mexico

TI 698 (Copan)	PRIMITIVE	Costa Rica

TI 981 Samsun	ORIENTAL	Brazil

TN 90	BURLEY	USA

Tomback	ORIENTAL	Unknown origin

Turkish Samsun	ORIENTAL	USA?

VA 355	FLUE CURED	USA

Wislika	FLUE CURED	Poland

YUN 85	FLUE CURED	Unknown origin

Zapatoca	CIGAR?	Colombia?

TI: tobacco introduction as numbered in the USA Nicotiana Germplasm collection.

TC: tobacco cultivars; included in this group is every cultivar, germplasm line, or genetic stock that has been registered with the Crop Science Society of America.

### LD decays in less than one cM along tobacco chromosomes

A total of 422 SSRs were used to scan the tobacco core collection at seven genomic regions located in different chromosomes (Table 5). The regions were selected based on marker density and their potential to harbor genes putatively important for crop improvement. The markers used had a density of 0.9 marker/cM, ranging from 0.6 on LG1 to 1.1 on LG17. The mean information index [41] varied from 1.68 for LG7 to 2.14 for LG17 (Table 5). Only 6.45%, 1.92%, and 6.06% of SSR markers, on LG1, LG7, and LG22, respectively, were found to be monomorphic. The lowest average number of alleles per locus was on LG7 (4.57 alleles per SSR), and the highest was on LG22 (7.8 per SSR; Table 5).

**Table 5:** Markers distribution and statistics concerning the selected genomic regions (standard errors in brackets)

Linkage Group	No. of SSRs	Interval spanned (cM)	**Mean Information index**	Mean Number. of alleles	% Polymorphic loci
1	62	40	1.70 (0.11)	6.1 (0.57)	93.55

2	38	40	2.00 (0.08)	5.7 (0.48)	100.00

7	50	52	1.68 (0.10)	4.57 (0.38)	98.00

12	71	65	1.99 (0.07)	6.28 (0,41)	100.00

17	55	57	2.14 (0.09)	7.6 (0.63)	100.00

22	66	74	1.98 (0.06)	7.8 (0.56)	98.00

23	80	70	1.74 (0.80)	5.24 (0.42)	100.00

Total	422	398		Avg. 6.18	

**Figure 4:**
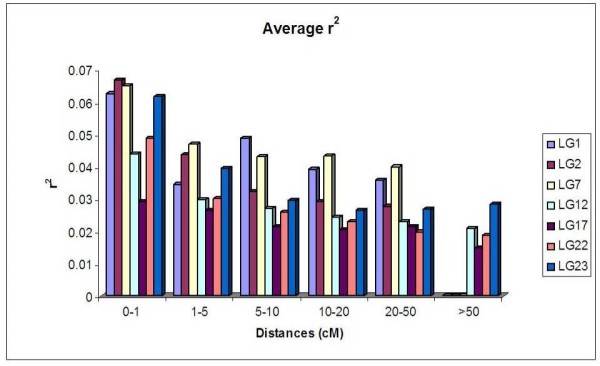
**Average long-range LD between SSR loci**. Pair-wise markers have been classified based on independently intermarker genetic distance. LD analysis was performed for each LG. For each class, the average *r^2 ^*value is reported.

**Figure 5:**
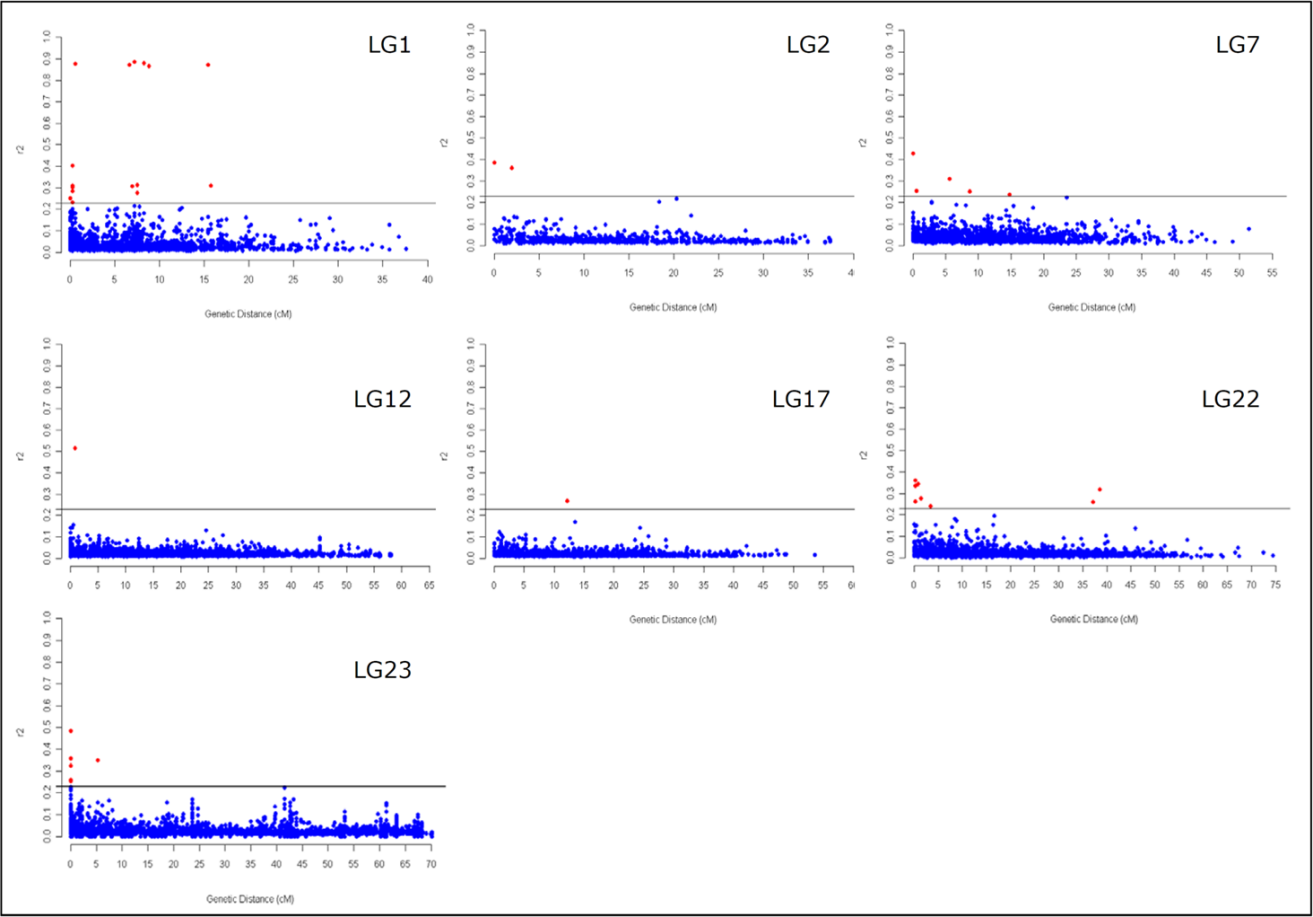
**Decay of LD (*r^2^*) as a function of genetic distance (cM)**. The *r^2 ^*= 0.23 threshold line is indicated.

The square root-transformed distribution of pairwise *r^2 ^*values of SSR loci mapping on different chromosomes (unlinked *r^2 ^*values) allowed us to set an appropriate threshold at a value of 0.23 beyond which LD values were considered significant. The value of 0.23 calculated for this LD threshold excluded most of the *r^2 ^*values of SSRs mapping to the same chromosomal region (linked *r^2 ^*values). Most of the marker pairs showing *r^2 ^*values above the threshold (on average 0.25% of the total pairwise values) were from loci mapping within a few cM (Figure 4), although outliers were also observed (Figure 5). On LG1, the pattern of LD rapidly decayed within less than 1 cM, although a total of 15 pairwise *r^2 ^*values of SSR loci mapping within 15 cM showed significant LD values (Figure 5). This block of significant LD encompasses 15 SSRs (PT20234n, PT50467, PT50754, PT50862, PT51015, PT51174, PT51438, PT51479, PT51966, PT54092, PT54727, PT54759, PT54767,PT54916 and PT61209) [33], 4 of which (PT51438, PT51479, PT51966 and PT54916) have an expected heterozygosity close to zero, while the remaining 11 showed an expected heterozygosity significantly lower than the mean expected heterozygosity of the SSRs of LG1 (data not shown). Taken together these data could point out that the 15 cM LD block revealed on LG1 was generated owing to a loss of allele diversity occurred in SSRs of this interval map. As expected, the trend was that LD decreased with genetic distance. On LG2, three pairs of markers had *r^2 ^*values exceeding 0.36. Along the same LG, a second region of *r^2 ^*values close to the threshold was observed in comparisons involving markers at a distance around 20 cM. On LG7, pairs of loci with significant LD were observed within 15 cM between markers, and close to the threshold at 25 cM. On both LG12 and LG17, only one pair of SSR loci had significant *r^2 ^*values. On LG12, the two loci were within 5 cM, while on LG17, the distance was around 12 cM. On LG22, eight pairwise *r^2 ^*values were above the threshold, two of which mapped within 35 cM. On LG23, five pairwise *r^2 ^*values were found between loci mapping within 5 cM.

The seven regions where the extent of LD was investigated encompass a significant sample of the total tobacco genome (12%; Gregor Bindler, personal communication). The extent (inter-marker distance in cM) of LD turned out to be limited to one cM or less, which is a very limited value, especially considering that in highly structured collections LD is expected to be overestimated [42]. Similar results were obtained for the sub-populations of tobacco based on tobacco type and identified with the clustering analysis, although, owing to the low number of genotypes, the significance of *P *values decreased with the exception of the "Flue-cured" sub-population (data not shown).

In tomato, the extent of LD was comparable to that of tobacco, but its magnitude was much higher [29]. In wheat, LD has a behavior similar to that of tobacco [43], with a low magnitude of LD detected over long segments of chromosomes. In a report concerning a different wheat collection, a genome-wide LD extending up to 10 cM with mean *r^2 ^*= 0.18 and much higher intrachromosomal *r^2 ^*values has been reported [25]. Similar levels of long-range LD extending over several cM have been found in self-pollinating species such as Arabidopsis [44] and barley [24]. The study of LD in maize carried out with inbred lines indicates its rapid decay within 1 cM up to values of *r^2 ^*<0.05, when assessed with intragenic SNPs, although much higher genome-wide LD levels were assessed using SSRs [45].

In general, long and local ranges of LD exhibited in a collection of crop genotypes depend on many factors, including the mating system as well as the evolutionary forces to which this crop was subjected. The global reduction of allelic diversity at whole genome loci generated by genetic bottlenecks tends to increase both long and local ranges LD [19,24]. On the contrary, selection fix one or few alternative alleles present in a population, causing a loss of allelic diversity only at the key loci under selection and at linked loci, a phenomenon known as genetic hitchhiking [21]. Consequently, selection can increases only the local range of LD at the target loci in which it acted [46]. The tobacco data discussed in this paper pointed out a 15 cM LD block in LG1 that could be a signature of selection as it is associated with a significant reduction of allelic diversity of SSRs (data not shown).

In turn, the usual division of the germplasm into alternative breeding groups facilitates the maintenance of alternative haplotypes in different gene pools, thus supporting high levels of LD between gene pools. The pattern of LD observed in tobacco is compatible with a structured population, i.e., strong bottlenecks occurred, particularly during breeding within the germplasm pool of a specific tobacco type, and our samples can be considered as derived from different populations. Thus, the observed LD has high values when considering inter-groups comparisons, but it is likely that the LD observed is in fact due to the structure of the collection analyzed.

In several instances, very distant pairs of markers with significant LD levels were observed, a finding which may be due to the low number of alleles at specific loci with minor alleles present at a very low frequency. We also observed "islands" of LD at a few positions along LGs. This could be a consequence of the lack of admixture between lines belonging to different types [34]. Alternatively, it could be the result of the presence of chromosomal translocations and/or inversions [47], or of the presence in the germplasm of genotypes with regions hosting hemizygous DNA stretches opposed to pairing and recombination [48].

In summary, the values of LD reported here have a similar pattern for all chromosomal regions tested, with few exceptions. These could be due to inaccuracies in the genetic distances reported in the linkage map, to misplacement of genetic marker loci, or to the low frequencies of specific alleles. The extent of LD measured in this work indicated that tobacco is not amenable to genome-wide association studies. Although it is true that a few marker pairs showed LD exceeding the threshold, the number of pairs was very low and did not exceed the number of false positive results expected for the significance testing procedure applied. Therefore, we concluded that the LD observed in the panel of tobacco accessions analyzed was insufficient to support the undertaking of subsequent long range association analyses, although the r^2 ^value detected are encouraging to carry association mapping when more molecular markers will become available.

A higher density of markers would probably make it possible to use LD to perform candidate-gene-based studies. We cannot exclude the possibility that by re-evaluating the LD using a higher density of markers and concentrating on shorter regions, we would observe a different situation, as LD has been shown to be population and locus specific [42].

## Conclusions

Our study demonstrated a low level of genetic diversity and a fast decay of LD in the seven regions that have been analyzed in the tobacco genome. Relatively recent evolutionary and breeding bottlenecks could account for the low levels of *Hd *revealed by molecular markers. Tobacco accessions were shown to cluster according to their market type, which, combined with a low level of admixture, is a further proof of the effectiveness of the conservative breeding programs. In our study, we have provided evidence of population substructure in tobacco and proposed, for the first time, a core collection. The level of LD observed was influenced by the structure of the population and by the recombinational history of the population, and it decayed in within very short intervals (less than 1 cM).

## Methods

### Plant material

A total of 312 tobacco accessions (Additional file 1) maintained at the Philip Morris International collection, Neuchatel (CH), were investigated in this study. Accessions were classified as described in Chaplin et al. [36]: "Burley" & "Maryland": 45 entries; "Flue-cured": 70; "Orientals": 77; "Cigar tobaccos" (filler, wrapper, binder): 36; "Primitives": 23; "Dark tobacco" & "Fire-cured": 22; "Other": 18 ("Perique", 1; "South American", 4; "Semi-oriental" 1; "Indonesian" and "other", 12). Twenty-one accessions were of unknown type.

The majority of accessions were originally obtained from the U.S. *Nicotiana *Germplasm collection in Oxford, NC (USA); the accessions used represent tobacco collected from or cultivated in 45 different countries. Seeds were germinated and grown under greenhouse conditions until plants reached a height of approximately 20 cm before DNA extraction.

### DNA extraction and genotyping

Leaves from 5 plants were pooled and genomic DNA was isolated from 6 mg of lyophilized material in 96-well microtube plates using Macherey Nagel^® ^NucleoSpin Plant II kit and following manufacturer's instructions. The quality and the concentration of the genomic DNA were assessed using electrophoretic analysis and Picogreen^® ^technology (Invitrogen, San Diego, CA), respectively. Genomic DNA was normalized at 20 ng/μL before genotyping.

All SSR loci considered in this study were amplified using a three-primer system for indirect labelling PCR fragments [49]. The amplification of SSR loci was carried out in 384-well plates (Applied Biosystems, Foster City, USA) in Eppendorf Mastercycler EPgradient thermalcyclers (Eppendorf, Hamburg, Germany). Each reaction was performed in 10 μl with the following mixture composition: 20 ng of DNA, 1.5 mM of MgCl_2_, 0.4 μM of the first primer, 0.2 μM of the second primer with M13 complementary tail, 0.2 μM of M13 fluorescent labelled primer, and 0.25 U of Taq HotStart DNA polymerase QIAGEN (Valencia, USA). The reactions were subjected to the following thermal protocol: after an initial denaturation step at 95 C for 15 min, amplification reactions were subjected to 11 cycles at 95 C for 30 s, 58 C for 45 s and 72 C for 90 s, decreasing annealing temperature by 0.7 C in each cycle. The reactions were further subjected to 29 cycles of 95 C for 30 s, 50 C for 45 s, and 72 C for 90 s. A final elongation step of 10 min was applied. 0.25 μL of amplification products, each of which was labeled with the four ABI dyes, was mixed with 10 μL of formamide, loaded in a ABI3730 DNA analyzer (Applied Biosystems), and analyzed through capillary electrophoresis.

Fragment analysis was carried out with GeneMapper^® ^4.0 software (Applied Biosystems, Foster City, USA) using stutter peaks of known sizes as internal controls. Automatic allele calls were subsequently assessed reviewing all electropherograms. Fragments of lengths not comparable to the control or with fluorescent intensities lower than 75 percent of the peak assumed as true allele were considered artefacts. Genotyping tables were exported as tab-delimited files and formatted in Microsoft Excel (Redmond, USA) to conduct phylogenetic and statistical analysis.

### Data analysis

Basic statistics (number of alleles detected at each locus, allelic and genotypic frequencies, call rate, heterozygosity, and PIC) were calculated using the *R *[50] and GenAlEx packages [51].

PHYLIP package gendist software [52] was used to calculate pairwise Cavalli-Sforza's genetic distances among the 312 tobacco accessions. Triangle matrix of pairwise genetic distances was subsequently formatted in NEXUS file to cluster the tobacco accessions with Neighbor-Joining using the SplitsTree4 software [53]. To better plot the resulting large clustering of tobacco accessions, a circular cladogram was generated with Dendroscope software [54].

To assess the population structure of the tobacco-sample accessions, a multivariate analysis and a heuristic method based on Bayesian clustering algorithms were utilized. Principal coordinate analysis (PCoA) was initially performed on the SSR data using the "ape" package in the *R *software. The clustering method based on the Bayesian-model implemented in the software program STRUCTURE [40] was used on the same data set to better detect population substructures. This clustering method is based on an algorithm that assigns genotypes to homogeneous groups in such a way that departure from neutral equilibrium is minimized among genotypes within each group, but it is absent among groups. The number of potential subpopulations varied from 2 to 10, and their contribution to the genotypes of the accessions was calculated based on 5x10^5 ^iteration burn-ins and 5x10^5 ^iteration sampling periods. Eventually, the most probable number (*K*) of subpopulations was identified following Evanno et al. [55].

Pairwise coefficients of kinship (*F_ij_*), a measure of relatedness between individuals *i *and *j *based on molecular markers, were calculated using SPAGeDi software [56]). Mean kinship (*mk*) coefficients were obtained averaging the pairwise kinship coefficients of each single accession with all other accessions of the whole collection [40]. In addition, the computation of mean kinship coefficients was restricted to pairwise kinship coefficients of accessions of the same tobacco type (*mk_i_*), as well as for all possible pairs of accessions of different tobacco types (*mk_p_*). In order to assess the higher level of relatedness of tobacco accessions of the same type, population kinship coefficients (*MK*) were calculated arithmetically averaging the *mk_i _*coefficients of tobacco accessions sharing the same tobacco type. Similarly, the level of relatedness of accessions of two different tobacco types was assessed averaging *mk_p _*coefficients calculated for all possible pairs.

### Core collection sampling

An algorithm was developed and implemented that allowed us to identify the least number of accessions capturing all of the alleles that were unique in the set of tobacco accessions. A first screening with one SSR marker was performed on a random sub-set of the accessions, followed by a pairwise comparison with the remaining accessions. Only accessions showing at least one unique allele were used in the following iterative analysis, leading to a list of accessions that represented all of the alleles. Because the group and the number of accessions in the final list can change, depending on the original order of the list, the accessions were randomly re-ordered and this process was repeated 2 x10^5 ^times. This method allowed for the selection of 60 genotypes. Additional methods were used to create three more lists of accessions, each with 60 genotypes showing the most extreme values of PCA, *mk *coefficients, and pairwise genetic distance. A fifth list of 60 genotypes was created by picking individuals with the highest values in the Q matrix of the STRUCTURE analysis. The five lists of genotypes were then merged and a consensus list of 89 genotypes was compiled.

### Analysis of linkage disequilibrium (LD)

The squared allele-frequency correlation *r^2^*, was calculated for all possible combinations of alleles to estimate the extent of LD in the core collection of tobacco accessions, using the software package TASSEL 2.01 [57]. The weighted average of *r^2 ^*values was obtained by further weighting for the corresponding allele frequencies. The significance of pairwise LD (*p*-value) among all possible pairs was also evaluated by TASSEL with the rapid permutation test.

To avoid the bias imposed by the usage of the squared-allele-frequency correlation *r^2 ^*in the presence of rare alleles, only alleles having a frequency larger than 0.1 were considered.

The square root of each pairwise *r*² among allelic variants of physically unlinked SSR loci was calculated. The 95^th ^percentile of this approximate normal distribution was assumed as the threshold of the *r*² value to declare the presence of LD among molecular markers [58].

## Authors' contributions

AF carried out the molecular genetics experiments, contributed to data analysis and drafted the manuscript. NB prepared plant material and contributed to data analysis. MDC contributed to data analysis. PF managed the high-throughput molecular markers platform. AS contributed to data analysis. PD drafted the manuscript. NVI contributed to data analysis and drafted the manuscript. CP managed the project, contributed to data analysis and drafted the manuscript. All authors read and approved the final manuscript.

## Acknowledgements

We wish to thank the personnel of the greenhouse at PMI and Gregor Bindler for their crucial help in providing the plant material. We also wish to thank Lynda Conroy for providing writing assistance.

## Supplementary Material

Additional file 1 **List of the 312 varieties**. Click here for file
