# Supplementary material for: Molecular diversity, population structure, and linkage disequilibrium in a worldwide collection of tobacco (Nicotiana tabacum L.) germplasm
Source: BMC Genet. 2012 Mar 21;13:18. doi: 10.1186/1471-2156-13-18 (PMC3342901; doi:10.1186/1471-2156-13-18)
Supplement: Additional file 1 — List of the 312 varieties. [file 1471-2156-13-18-S1.PDF]

List of the 312 varieties

1	TI404
2	VA359
3	TC189
4	TI981
5	Mtra88
6	NC 2000
7	Havana 322
8	TI 193
9	Kutsaga 35
10	Pina
11	DF Black Mammoth 2
12	DF Black Mammoth
13	TR Madole
14	Chileno Grande Amarellino
15	Dynes
16	NC 95
17	ITB 30
18	Speight 70
19	K 394
20	Oxford 207
21	Cordoba 2
22	K346
23	Mc Nair 373
24	pvh03
25	bhavya
26	Coker 371 Gold
27	K 326
28	TC365
29	Speight 179
30	K149
31	K149-2
32	NC 55
33	Cetarsa 26
34	RG17
35	Speight G28
36	Golta
37	Speight 168
38	Speight 172
39	Pelorest
40	Hicks Broaleaf
41	Swarna
42	Lonibow
43	Rhayva
44	Iamatoa
45	Little crittenden
46	Golta
47	Dixie Bright 244
48	Coker 178
49	Mc Nair 944

50	Coker 347
51	K 326
52	Cetarsa 19
53	Cash
54	Oxviz
55	DF Shirey
56	Little crittenden
57	Greenwood little wood
58	VA 309
59	DF Lizard Tail
60	DF Lizard Tail 2
61	TN D950
62	TN D950-2
63	McNair 135
64	Cordoba
65	Vinit
66	MAFC 5
67	Mevesi 17
68	ITB 620
69	Rama
70	Dixie Bright 27
71	K 346
72	K 358
73	BRIK2
74	Yun 85
75	K 399
76	Wislika
77	NC 89
78	K 394 2
79	MAFC 34
80	LAFC53
81	Kolitschestwo
82	RG8
83	Perique TC 556
84	VA 355
85	DF Greenwood
86	Itzepeque
87	TI 698 Copan
88	Aparecido
89	Virginia
90	TI 613
91	TI 592
92	TI 189
93	Criollo Salteno
94	TI 675
95	Criollo
96	TI 77
97	Criollo colorado
98	TI 1070
99	TI 1068

100	TI 1031
101	Havana 142
102	TI 1077
103	Zimen Spanish
104	Ottawa 705
105	Guacharo oriente
106	Havana 426
107	Low nic german
108	Lysistrata
109	Saturn 280
110	TI 501
111	Mont calme jaude
112	Turuki
113	TI 946
114	TI 470
115	Ilopango
116	Tabaco negro
117	Tabaco Negro 2
118	Malopolanin
119	CBI
120	CBI
121	CBI
122	CBI
123	CBI
124	CBI
125	CBI
126	Golta
127	Zambo galado
128	TI 102
129	Tabaco Blanco
130	TI 698
131	Habano
132	TI 1117
133	Ambalema
134	Gober peloes
135	TI 437
136	KY 8959
137	Palmita
138	Zapatoa
139	TI 535
140	Coltabaco 23
141	Coltabaco 1A
142	Coltabaco 2A
143	Elasona Kabakoulak
144	Kabakoulak Zagliveti
145	Lattaquie
146	Malatya
147	Bahce
148	karabaglar
149	Izmir_Akhisar

150	Izmir_Edge_64
151	Izmir_Gavurkoy
152	Izmir_Gordes
153	Vena amarilla
154	Kirkagac
155	Mendek
156	SImmaba
157	Vorstenlanden
158	S-1 ATC89
159	Okinawa
160	Mihara
161	TI 1275
162	N Gana N Gana
163	TI 1309
164	Florida sumatra
165	TI 1568
166	Bonanza
167	Suknumi 959
168	Tasouoa
169	Turkish Samsun
170	Turkish tropizoid
171	Adiyaman
172	Samsun maden
173	Bafra
174	Yaka 125
175	Yaka 87
176	Djebal Pobeda
177	Djebal 81
178	Saade
179	Yaka 49
180	Dubek 7
181	Charmanilisjska basma
182	Otja 110
183	BMV617
184	Krumovgrad
185	Barinas
186	TI 1271
187	TI 1269
188	Tomback
189	Djebel_81
190	Dejbel_38
191	Sendinii
192	Krumovgrad_988
193	Krumovgrad_58
194	Turkish_variotic
195	Xanthi_Balanos
196	BM-V5-5-2_MEGA_PISTO
197	BM_V_3_10_ORGANi
198	BM_V_10_12_LIKIO
199	Yayladag

200	Komotini_Balanos
201	Basma_Llovina
202	Basma_Nigrita
203	Tobacco seed unkn
204	Basmak
205	Yaka 23
206	BM-V-9-14_GRATINI
207	BM-V-1-20_MIKRO_PISTO
208	BM-V-5-5AGIOCHORI
209	BM-V-8-14_AGRAS
210	Prilep_23
211	Prilep_10
212	BM-V-2-_KOMOTINI
213	Otja_87
214	Basma_Komotini
215	Basma_Drama_B84_31
216	Basma Xanthi bx24
217	Basma_Komotini_BX81BXK
218	Prilep 153
219	Yayladag
220	Prilep 80
221	FT 157
222	Tomback Iran
223	BP0316
224	Deli Sumatra
225	Sumatra congo
226	Samsun
227	Samsun Batra
228	Samsun TC 541
229	Bitlis 90
230	Izmir edge 64
231	Ternopilsjiiy
232	Nevrokop_261
233	TI 112
234	DF Greenwood 2
235	Barbasco
236	Amarillo planchado
237	DAC Mata Fina Wrapper
238	Piyanguy Minas
239	Piloto Cubano
240	BP 035
241	KY Black
242	Criollo Especial
243	Criollo Especial-1
244	Connecticut TC184
245	Big Cubain
246	Baiano
247	MPESKQ
248	Florida 301
249	Orinoco PMS22

250	TI 81 Orinoco
251	Colorado
252	Stamm 160
253	Tabaquillo
254	TI 606
255	Palmira
256	Chiricano
257	ULT 661
258	ULT 526
259	ULT655
260	KY 10
261	B841052
262	Dreta
263	Bu Slovak
264	Palagi
265	TC575
266	Apia
267	Golden Burley
268	Chocoa
269	Machudi
270	Dungowad
271	Maryland 609
272	Maryland 402
273	Banket A1
274	TN88
275	Sevilla 6
276	Granada 11
277	Sevilla 12
278	TN 90
279	KY908
280	Jarandilla 1
281	ITB2604
282	KY907
283	BB16A
284	ITB501
285	ITB218
286	TN90-2
287	KBM33
288	TI 216
289	ky 15
290	kbm 20
291	Kentucky 14
292	VA510
293	BU 64
294	Baldio Vera
295	Saplak
296	Havana 503
297	Bu21
298	Burley 49
299	Jupiter

300	BS92
301	Payta
302	TI 1406
303	KDH-960_TC_466
304	TI246
305	KDH959
306	ULT308
307	Amarellinho
308	TI1078
309	Chileno Grande Colorado
310	CBI
311	Yellow twist bud
312	Yellow twist bud2

CBI: confidential business information
